# Sex differences in obesity-induced renal lipid accumulation revealed by lipidomics: a role of adiponectin/AMPK axis

**DOI:** 10.1186/s13293-023-00543-6

**Published:** 2023-09-28

**Authors:** Florian Juszczak, Louise Pierre, Morgane Decarnoncle, Inès Jadot, Blanche Martin, Olivia Botton, Nathalie Caron, Jonas Dehairs, Johannes V. Swinnen, Anne-Emilie Declèves

**Affiliations:** 1 Laboratory of Metabolic and Molecular Biochemistry, Faculty of Medicine and Pharmacy, Research Institute for Health Sciences and Technology, University of Mons (UMONS), Mons, Belgium; 2 Molecular Physiology Research Unit (URPhyM), Namur Research Institute for Life Sciences (NARILIS), University of Namur (UNamur), Namur, Belgium; 3 Biochemistry and Cellular Biology Research Unit (URBC), Namur Research Institute for Life Sciences (NARILIS), University of Namur (UNamur), Namur, Belgium; 4 Laboratory of Lipid Metabolism and Cancer, Department of Oncology, KU Leuven, Leuven, Belgium

**Keywords:** Sex difference, Chronic kidney disease, Obesity, Renal lipids, Adiponectin, AMPK

## Abstract

**Background:**

Sex differences have been observed in the development of obesity-related complications in patients, as well as in animal models. Accumulating evidence suggests that sex-dependent regulation of lipid metabolism contributes to sex-specific physiopathology. Lipid accumulation in the renal tissue has been shown to play a major role in the pathogenesis of obesity-induced kidney injury. Unlike in males, the physiopathology of the disease has been poorly described in females, particularly regarding the lipid metabolism adaptation.

**Methods:**

Here, we compared the lipid profile changes in the kidneys of female and male mice fed a high-fat diet (HFD) or low-fat diet (LFD) by lipidomics and correlated them with pathophysiological changes.

**Results:**

We showed that HFD-fed female mice were protected from insulin resistance and hepatic steatosis compared to males, despite similar body weight gains. Females were particularly protected from renal dysfunction, oxidative stress, and tubular lipid accumulation. Both HFD-fed male and female mice presented dyslipidemia, but lipidomic analysis highlighted differential renal lipid profiles. While both sexes presented similar neutral lipid accumulation with obesity, only males showed increased levels of ceramides and phospholipids. Remarkably, protection against renal lipotoxicity in females was associated with enhanced renal adiponectin and AMP-activated protein kinase (AMPK) signaling. Circulating adiponectin and its renal receptor levels were significantly lower in obese males, but were maintained in females. This observation correlated with the maintained basal AMPK activity in obese female mice compared to males.

**Conclusions:**

Collectively, our findings suggest that female mice are protected from obesity-induced renal dysfunction and lipotoxicity associated with enhanced adiponectin and AMPK signaling compared to males.

## Highlights

- HFD female mice are protected from ectopic lipid accumulation in the renal tubules compared to males, despite similar body weight gain.
- Lipidomic analysis revealed sex differences in renal lipid profiles.
- HFD female mice are protected from renal phospholipid accumulation and lysosomal dysfunction compared to HFD male mice.
- HFD males specifically present increased levels of very-long-chain ceramides in the kidney and decreased AMPK activity.
- HFD females may be protected from renal injury by higher levels of adiponectin, which correlates with higher AMPK activity in the kidney.

## Plain language summary

Obesity-related complications can differ between men and women due to sex-specific differences in how fats are handled. Here, we studied the effects of high-fat diet on the kidneys of male and female mice. We found that despite gaining similar weight, obese female mice were better protected against insulin resistance, liver fat accumulation, and kidney damage caused by obesity than males. In particular, female mice were protected against lipid accumulation in the kidneys. We further analyzed the lipid profile in the kidneys of both male and female mice and observed differences in the amount and nature of the accumulated lipids. Male mice had increased levels of specific lipids, which may contribute to their higher risk of kidney damage. In contrast, female mice showed better lipid metabolism adaptation, which helped to protect their kidneys. This study also revealed an association between higher levels of the protein hormone adiponectin and higher activity of the cellular energy master regulator protein AMPK in obese females. These proteins may help prevent obesity-induced kidney damage. In obese males, these protective proteins are reduced and are associated with kidney damage. In conclusion, this study suggests that female mice are naturally shielded from obesity-induced kidney damage and lipid accumulation in the kidneys. Obesity in males is associated with the presence of potentially toxic lipids and dysregulated renal metabolism. Understanding these sex-related differences in obesity-related complications could lead to better management and treatment of kidney problems in both men and women.

## Introduction

The incidence of obesity has dramatically increased over the last few years and is expected to reach 50% by 2030 [1]. Obesity is a major risk factor for chronic kidney disease (CKD), and is the second most important predictor of end-stage renal disease [2]. Ectopic lipid accumulation in proximal tubular cells (PTC) have also been observed, indicating that lipotoxicity play a role in the development and progression of CKD [3]. Notably, sex differences have been observed in the development of obesity and obesity-related complications in both animal models and patients [4–8]. Men and women differ in the underlying pathophysiology of CKD and its associated complications [9]. Epidemiological studies on the global burden of CKD highlighted a higher prevalence in women, whereas mortality is higher in men [10]. Although recent studies have provided some insights into the sexual dimorphism of obesity-induced CKD, there remains a critical need to gain a better understanding of the physiological sex-specific characteristics of this disease.

Mice fed a HFD have been widely used to study obesity-related disorders [11]. We and others have previously demonstrated the renal consequences of obesity in this animal model [2, 12–16]. HFD-fed male mice developed human characteristic features of obesity-induced CKD including glomerulosclerosis, tubulointerstitial fibrosis associated with albuminuria and proteinuria [17]. In addition, obesity-induced CKD is characterized by ectopic lipid depositions in renal PTC both in rodents and humans, leading to renal lipotoxicity [12–14, 18–20]. Indeed, dysregulated renal lipid metabolism, particularly fatty acid oxidation impairment, has been discovered to play a major role in CKD development and progression [21]. Particularly, accumulation of lysosomal phospholipids was demonstrated in PTC of obese male mice, which is associated with lysosomal dysfunction, impaired autophagic flux, mitochondrial dysfunction, and inflammasome activation [13, 22, 23]. Even if the renal consequences of obesity are well characterized in males, to date, there are still no reliable data in females. To note, we and others demonstrated the pivotal role of AMPK in the development of obesity-induced CKD [24–29]. AMPK is a ubiquitous heterotrimeric kinase considered as an energetic sensor responding to changes in the intracellular AMP/ATP ratio [30]. Metabolic stresses impairs AMPK activity in the renal cortex of obese and diabetic male mice [3, 25]. In addition, AMPK activation prevents renal inflammation, oxidative stress and improves mitochondrial dysfunction in several male animal models of CKD [3, 14, 31, 32]. Moreover, adiponectin has been shown to enhance AMPK activity in various tissues, including the kidney [33, 34]. However, in diabetes, adiponectin production is decreased, leading to impaired AMPK activity in the renal cortex of diabetic male mice [26, 35]. Adiponectin deficiency has been associated with the development of insulin resistance and metabolic dysfunction in various tissues, including the kidney. Adiponectin activates AMPK and promotes fatty acid oxidation [36]. Studies have shown that females tend to have higher levels of adiponectin compared to males, which may contribute to sex differences in lipid metabolism [37, 38]. Given the limited understanding of the effects of obesity on CKD in females, the present study seeks to comprehensively characterize the sex-specific consequences of obesity on kidney function in HFD-fed mice. Here, we hypothesized that HFD triggers differential accumulation of lipid species in the kidneys of male and female mice, explaining the differences in the development and progression of obesity-induced CKD. In this study, we used targeted lipidomic analysis to quantify the sexual dimorphism in renal lipid accumulation. Moreover, the AMPK–adiponectin axis was investigated to further highlight its implications in renal lipid metabolism and CKD outcomes.

## Materials and methods

### Animals

The study conformed to the APS Guidelines for the Care and Use of Animals and was approved by the Animal Ethics Committee of the University of Namur. The experiments were conducted on C57Bl/6J male and female mice (Janvier Labs, Le Genest Saint-Isle, France) that were housed in cages with free access to food and water. The mice were maintained at 35–40% relative humidity and a temperature of 20–23 °C with a 12:12 h light–dark cycle. Over a 16-week period, 8-week-old mice (*n* = 6 per sex and per treatment) were randomized to either a LFD (10% of total calories from fat; D12450J, Research Diets, New Brunswick, NJ, USA) or HFD (60% of total calories from fat; D12492, Research Diets, New Brunswick, NJ, USA). Body weight (BW) was measured every 4 weeks and 24-h urine collection was performed at the end of the study using metabolic cages. Fasting blood glucose concentration (One Touche Vita, LifeScan Inc., Milpitas, CA) was measured from the tail vein. At week 16, mice were anesthetized after an overnight fast with a solution of ketamine (Nimatek^®^, Eurovet Animal Health, Blabel, The Netherlands, 80 mg/kg b.w.) and medetomidine (Domitor^®^, Orion Pharma, Espoo, Finland, 0.5 mg/kg b.w.), and blood was collected by intracardiac puncture. Blood samples were collected and centrifuged at a high speed for 20 min at 4 °C. The plasma was collected and stored at − 80 °C until further use. The kidneys, liver, and heart were removed, weighed, and reported to tibia length in order to normalize the data, as previously reported [14]. A portion of the collected organs was fixed in Duboscq-Brazil solution, while the remaining tissues were frozen in liquid nitrogen and stored at − 80 °C for further analysis.

### Urine collection and urinary markers analyses

After 16 weeks of feeding, mice were placed for 24-h urine collection in metabolic cages that provide ad libitum access to food and water and allow refrigeration of urine samples. Urine was subsequently stored at − 20 °C. Urinary albumin and creatinine levels were measured using a mouse Albuwell ELISA kit and Creatinine Companion kit (Exocell, Philadelphia, PA, USA). Total proteinuria was quantified using the Bradford method based on the absorbance of the Coomassie Brilliant Blue dye. As an index of oxidative stress, urine samples were analyzed for hydrogen peroxide using the Amplex red assay (Thermo Fisher Scientific, Waltham, MA, USA) following the manufacturer’s instructions. All urinary marker values were normalized to the urinary creatinine concentration.

### Biochemical assays

Plasma leptin and adiponectin (full-length form of adiponectin) concentrations were determined by ELISA (Mouse/rat leptin immunoassay Quantikine ELISA, Mouse adiponectin/Acrp30 Immunoassay Quantikine ELISA, R&D Systems Europe, Abingdon, UK). Plasma insulin levels were determined by ELISA using the rat/mouse insulin ELISA kit (Merck, Darmstadt, Germany). The homeostasis model assessment (HOMA-IR) for the insulin resistance index was determined using a calculator available from the Oxford Center for Diabetes, Endocrinology, and Metabolism (https://www.dtu.ox.ac.uk/homacalculator/). Colorimetric enzymatic tests were performed to measure plasma cholesterol levels (Diasys, Diagnostic System, Holzheim, Germany) and plasma non-esterified fatty acid (NEFA) levels (Wako Pure Chemical Industries, Ltd., Osaka, Japan), following the manufacturer’s instructions.

### Histology and morphological analyses

Five-μm paraffin-embedded kidney sections were stained with Periodic Acid Schiff (PAS), Hemalun, and Luxol Fast Blue to assess morphological alterations. Morphometry of kidney sections was performed as previously reported [13]. Briefly, the frequency of tubules containing vacuolated cells was evaluated using a semi-quantitative single-blind analysis. To standardize the evaluation procedure, an additional lens engraved with a square grid was inserted into one of the microscope’s eyepieces. For each paraffin section, 10 square fields (0.084 mm^2^/field) were observed at × 400 magnification. Ten randomly selected areas of each cortex kidney section were analyzed using the ImageJ software. Paraffin-embedded liver sections were stained with hematoxylin and eosin and steatosis was graded as described by Ryu et al. [39].

### Immunohistochemistry

Five-μm paraffin-embedded kidney sections were dewaxed and rehydrated, followed by microwave pre-treatment in 1 mM EDTA buffer to unmask antigens present in the renal tissue. Endogenous peroxidase activity was removed by incubation with 3% H_2_O_2_ for 10 min and blocking with 10% normal goat serum. Sections were incubated with a primary antibody against LAMP-1 (Abcam, Cambridge, UK) overnight at 4 °C. After rinsing in TBS, slides were exposed for 30 min with SignalStain^®^ Boost IHC Detection Reagent (Cell Signaling, Danvers, MA, USA), and bound peroxidase activity was detected using a DAB kit (Agilent DAKO, Heverlee, Belgium). Counterstaining was performed using Hemalun and Luxol Fast Blue. The evaluation of the relative positive area was performed on one section per experimental animal. For each section, ten square fields (0.084 mm^2^/field) were observed at 400 × magnification in each renal zone. The relative area occupied by positive staining was expressed as a percentage.

### Quantitative real-time polymerase chain reaction (PCR)

Frozen kidney cortex was homogenized and total RNA was extracted using TRIzol (Sigma-Aldrich, St. Louis, MO, USA) and treated with DNAse (Promega, Madison, WI, USA). The total RNA concentration was measured using a NanoDrop spectrophotometer (NanoDrop 1000, Thermo Fisher Scientific, Waltham, MA, USA). Transcript-specific primers were generated based on the mouse sequences from GenBank. The NCBI Primer BLAST was used to ensure the specificity of the primers for each target. All primer pairs were analyzed for their dissociation curves and melting temperatures. Real-time quantitative PCR was performed to quantify the mRNA levels of *AdiporR1*, *Lamp1*, *Cathepsin D*, *p62*, *CerS2*, *CerS5*, *CerS6*, *Acer2*, *Acer3*, and *18S* as housekeeping gene (Table 1). Briefly, 2 μg of total RNA was reverse-transcribed using MLV reverse transcriptase (Promega, Madison, WI, USA) for 1 h at 70 °C. Quantitative PCR amplification was performed using SYBR Green Master Mix (Roche, Belgium) and Prism 7300 Real-Time PCR Detection System (Applied Biosystems, CA, USA). Mean fold changes were calculated by averaging duplicate measurements for each gene. The relative gene expression was calculated using the 2^−ΔΔCT^ method.

**Table 1:** Primer sequences for RT-qPCR analysis of mRNA expression

Gene		Primer sequences (5′–3′)
CerS2	Fw	ATGCTCCAGACCTTGTATGACT
	Rv	CTGAGGCTTTGGCATAGACAC
CerS5	Fw	CGGGGAAAGGTGTCTAAGGAT
	Rv	GTTCATGCAGTTGGCACCATT
CerS6	Fw	GATTCATAGCCAAACCATGTGCC
	Rv	AATGCTCCGAACATCCCAGTC
Acer2	Fw	GTGTGGCATATTCTCATCTG
	Rv	TAAGGGACACCAATAAAAGC
Acer3	Fw	TGTGATTCACTGAGGAACTTTCG
	Rv	AGAAACTTCACTTTTGGCCTGTA
AdipoR1	Fw	TTTGCCACTCCCAAGCAC
	Rv	ACACCACTCAAGCCAAGTCC
Lamp1	Fw	CAGCACTCTTTGAGGTGAAAAAC
	Rv	ACGATCTGAGAACCATTCGCA
CathepsinD	Fw	CTCAAAGGCCCCATCACCAA
	Rv	TGCCGTTCTTCACATAGG
p62	Fw	GATGTGGAACATGGAGGGAAGAG
	Rv	AGTCATCGTCTCCTCCTGAGCA

### Western blot analysis

Proteins were extracted from renal cortex tissues using Cell Lysis Buffer (Cell Signaling, Danvers, MA, USA) with phosphatase and protease inhibitor cocktail (Thermo Fisher Scientific, Waltham, MA, USA) at 4 °C followed by centrifugation at 14,000 × g for 15 min at 4 °C. Protein concentrations were quantified by Pierce BCA assay kit (Thermo Fisher Scientific, Waltham, MA, USA) and then 20 µg of total lysate were separated by SDS-PAGE 12% and transferred onto nitrocellulose membranes. Following blocking step in 5% BSA for 1 h, the membranes were incubated with primary antibodies against phosphorylated AMPK (P-AMPK), AMPK (Cell Signaling, Danvers, MA, USA), AdipoR1 (Abcam, Cambridge, UK) or β-actin (Thermo Fisher Scientific, Waltham, MA, USA) overnight at 4 °C and then with secondary antibodies (Li-Cor Biosciences, Lincoln, NE, USA) for 1 h at room temperature. Antibodies were diluted in Odyssey Blocking Buffer TBS containing 0.1% Tween20. Proteins were visualized and quantified using the Odyssey^®^ imaging system (Li-Cor Biosciences, Lincoln, NE, USA).

### Lipidomic analysis

*Lipid extraction* Tissue lysates containing 10 μg of DNA were homogenized in 700 μl of water with an handheld sonicator and were mixed with 800 μl HCl(1 M):CH3OH 1:8 (v/v), 900 μl CHCl3, 200 μg/ml of the antioxidant 2,6-di-tert-butyl-4-methylphenol (BHT; Sigma-Aldrich, St. Louis, MO, USA) and 3 μl of SPLASH^®^ LIPIDOMIX^®^ Mass Spec Standard (#330707, Avanti Polar Lipids, Birmingham, AL, USA). After vortex and centrifugation, the lower organic fraction was collected and evaporated using a Savant Speedvac spd111v (Thermo Fisher Scientific, Waltham, MA, USA) at room temperature and the remaining lipid pellet was stored at − 20 °C under argon.

*Mass spectrometry* (*MS*) Lipid pellets were reconstituted in 100% ethanol and analyzed by liquid chromatography electrospray ionization tandem mass spectrometry (LC-ESI/MS/MS) on a Nexera X2 UHPLC system (Shimadzu) coupled with hybrid triple quadrupole/linear ion trap mass spectrometer (6500 + QTRAP system; AB SCIEX). Chromatographic separation was performed on a XBridge amide column (150 mm × 4.6 mm, 3.5 μm; Waters) maintained at 35 °C using mobile phase A [1 mM ammonium acetate in water–acetonitrile 5:95 (v/v)] and mobile phase B [1 mM ammonium acetate in water–acetonitrile 50:50 (v/v)] in the following gradient: (0–6 min: 0% B → 6% B; 6–10 min: 6% B → 25% B; 10–11 min: 25% B → 98% B; 11–13 min: 98% B → 100% B; 13–19 min: 100% B; 19–24 min: 0% B) at a flow rate of 0.7 ml/min which was increased to 1.5 ml/min from 13 min onwards. Sphingomyelins (SM), cholesterol esters (CE) and ceramides (CER) were measured in positive ion mode with a precursor scan of 184.1, 369.4, 264.4, respectively. Triacylglycerides (TG), diacylglycerides (DG) and monoacylglycerides (MG) were measured in positive ion mode with a neutral loss scan for one of the fatty acyl moieties. Phosphatidylcholines (PC), phosphatidylethanolamines (PE), phosphatidylglycerols (PG), phosphatidylinositols (PI) and phosphatidylserines (PS) were measured in negative ion mode by fatty acyl fragment ions. Lipid quantification was performed by scheduled multiple reactions monitoring, the transitions being based on the neutral losses or the typical product ions as described above. The instrument parameters were as follows: Curtain Gas = 35 psi; Collision Gas = 8 a.u. (medium); IonSpray Voltage = 5500 V and − 4500 V; Temperature = 550 °C; Ion Source Gas 1 = 50 psi; Ion Source Gas 2 = 60 psi; Declustering Potential = 60 V and − 80 V; Entrance Potential = 10 V and − 10 V; Collision Cell Exit Potential = 15 V and − 15 V.

The following fatty acyl moieties were taken into account for the lipidomic analysis: 14:0, 14:1, 16:0, 16:1, 16:2, 18:0, 18:1, 18:2, 18:3, 20:0, 20:1, 20:2, 20:3, 20:4, 20:5, 22:0, 22:1, 22:2, 22:4, 22:5 and 22:6 except for TGs which considered: 16:0, 16:1, 18:0, 18:1, 18:2, 18:3, 20:3, 20:4, 20:5, 22:2, 22:3, 22:4, 22:5, 22:6.

*Data analysis* Peak integration was performed with the MultiQuant™ software version 3.0.3. Lipid species signals were corrected for isotopic contributions (calculated with Python Molmass 2019.1.1), quantified based on internal standard signals and adheres to the guidelines of the Lipidomics Standards Initiative (LSI) (level 2 type quantification as defined by the LSI).

### Statistical analysis

The results are presented as the mean ± SEM. The level of statistical significance was set at *p* < 0.05. Analyses were performed using Prism GraphPad Software version 6 (San Diego, CA, USA). Differences were analyzed using two-way ANOVA to examine the overall effects of diet, sex, and interaction (see detailed statistical analysis in Table 2). In cases of significant ANOVA effects, post hoc comparisons were performed using Tukey’s multiple comparisons test to determine the significance between groups when appropriate. For lipidomic data, multiple t-tests with *p*-values corrected for multiple comparisons using the Bonferroni method were performed within each sex-matched group to analyze the FA profile for each lipid class. Partial least squares discriminant analysis (PLS-DA) and heat map visualization were performed using MetaboAnalyst 4.0 (http://www.metaboanalyst.ca).

**Table 2:** Results from two-way ANOVA statistical analysis

Figure#	Measurement	Diet				Sex				Interaction		
		DFn, DFd	F	*p*		DFn, DFd	F	*p*		DFn, DFd	F	*p*
1	Plasma leptin (ng/mL)	1.20	64.52	**< 0.0001**		1.20	8.31	**0.0092**		1.20	3.98	0.0597
1	Fasting blood glucose (mg/dL)	1.20	14.15	**0.0004**		1.20	18.02	**0.0012**		1.20	2.35	0.1409
1	Plasma insulin level (ng/mL)	1.17	8.17	**0.0109**		1.17	1,74	0.2043		1.17	1.17	0.2938
1	HOMA-IR	1.16	27.11	**< 0.0001**		1.16	13.79	**0.0019**		1.16	6.75	**0.0194**
1	Plasma FFA (mg/dL)	1.17	26.63	**< 0.0001**		1.17	4.36	**0.0521**		1.17	0.53	0.4762
1	Plasma cholesterol (mg/dL)	1.18	25.22	**< 0.0001**		1.18	3.41	**0.0813**		1.18	0.26	0.6131
1	Hepatic steatosis score	1.20	43.44	**< 0.0001**		1.20	7.98	**0.0105**		1.20	5.99	**0.0237**
2	UACR (µg/mg Cre)	1.17	12.82	**0.0023**		1.17	0.39	0.5406		1.17	0.24	0.6305
2	Urinary protein (mg/mg Cre)	1.17	9.43	**0.0069**		1.17	18.53	**0.0005**		1.17	2.97	0.1028
2	Urinary H_2_O_2_ (nmol/mg Cre)	1.20	19.73	**0.0003**		1.20	49.84	**< 0.0001**		1.20	10.82	**0.0037**
2	Vacuolated tubules (/mm^2^)	1.20	16.36	**0.0006**		1.20	11.55	**0.0029**		1.20	10.99	**0.0035**
6	% of LAMP1^+^ area	1.20	10.98	**0.0044**		1.20	12.61	**0.0027**		1.20	6.94	**0.0181**
6	LAMP1 mRNA expression	1.20	8.19	**0.0093**		1.20	23.69	**< 0.0001**		1.20	1.95	**0.0177**
6	CathepsinD mRNA expression	1.20	8.36	**0.0087**		1.20	12.48	**0.0020**		1.20	0.28	0.6008
6	p62 mRNA expression	1.20	5.74	**0.0260**		1.20	37.47	**< 0.0001**		1.20	0.19	0.6700
7	CerS2 mRNA expression	1.18	7.07	**0.0160**		1.18	44.36	**< 0.0001**		1.18	2.626	0.1225
7	CerS5 mRNA expression	1.19	23.85	**0.0001**		1.19	29.48	**< 0.0001**		1.19	0.01	0.9307
7	CerS6 mRNA expression	1.19	25.34	**< 0.0001**		1.19	46.65	**< 0.0001**		1.19	1.702	0.2076
7	Acer2 mRNA expression	1.19	18.97	**0.0004**		1.19	8.23	**0.0098**		1.19	3.93	0.0622
7	Acer3 mRNA expression	1.19	12.83	**0.002**		1.19	17.89	**0.0005**		1.19	1.09	0.3086
8	P-AMPK to AMPK ratio	1.19	9.29	**0.0066**		1.19	66.32	**< 0.0001**		1.19	3.51	0.0764
8	Plasma adiponectin (µg/mL)	1.20	0.16	0.6946		1.20	12.67	**0.0020**		1.20	0.84	0.3717
8	AdipoR1/ β-actin	1.19	1.145	0.2981		1.19	5.521	**< 0.0001**		1.19	0.2969	0.0822
8	AdipoR1 mRNA expression	1.16	6.12	**0.0242**		1.16	21.36	**0.0002**		1.16	4.5	**0.0490**

The table provides degrees of freedom, F and *p* scores for two-way ANOVA analyses. Significant *p*-values are displayed in blod

## Results

### Sexual dimorphism in metabolic parameters in response to HFD

**Fig. 1:**
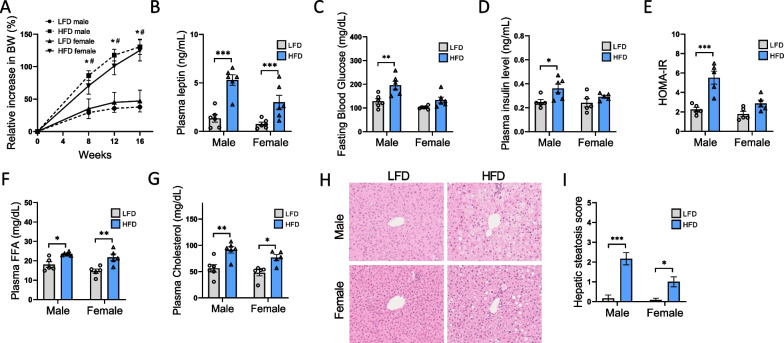
Sex differences in metabolic parameters in response to HFD. Male and female C57BL/6J mice were fed with either HFD or LFD for 16 weeks. **A** Relative increase in BW. Statistical analyses were performed using two-way ANOVA followed by Tukey post-test. *p < 0.05 HFD vs LFD male and ^#^*p* < 0.05 HFD vs LFD female. *n* = 6 in each group. **B** Plasma leptin level. **C** Fasting blood glucose and **D** plasma insulin levels were assessed in male and female mice fed a LFD or a HFD at week 16. **E** HOMA-IR index calculation. **F** Plasma FFA and **G** cholesterol levels. **H** Representative photomicrographs (original magnification ×400) of H&E staining illustrating hepatic steatosis from liver sections in males and females fed a LFD or a HFD. **I** Semiquantitative grading of hepatic steatosis in each group. Statistical analyses were performed using two-way ANOVA followed by Tukey post-test. *p < 0.05; **p < 0.01; ***p < 0.001. Data are presented as means ± SEM

To determine the differential effects of sex on diet-induced obesity, 8-week-old male and female C57Bl/6J mice were fed either a HFD or a LFD for 16 weeks. BW was measured over time and is shown in Fig. 1A as the relative BW gain (in % of the corresponding BW at the beginning of the protocol) to overcome sex differences. A similar relative increase in BW was measured in both the female and male HFD feeding groups throughout the experimental protocol. Both sexes also presented identical BW gains in the LFD-fed groups. Moreover, plasma leptin concentration was evaluated as a marker of diet-induced obesity. Leptin is an adipokine that correlates with body fat mass and increases with obesity [40]. Fig. 1B reveals a strong impact of the HFD on leptin concentration, as demonstrated by the two-way ANOVA analysis (P_Diet_ < 0.0005). Moreover, post hoc comparisons revealed that plasma leptin levels were significantly higher in both HFD-fed male and female mice than in LFD-fed mice. However, the plasma leptin levels were significantly lower in females than in males (P_Sex_ < 0.0005). HFD-induced obesity in mouse models has been associated with insulin resistance. Therefore, we investigated the relationship between sex, diet, and glucose metabolism in HFD-fed mice. As illustrated in Fig. 1C, D, HFD-fed females were not affected by hyperglycemia and hyperinsulinemia compared with males. HOMA-IR calculation revealed that HFD induced insulin resistance in males (5.52 ± 0.67), but not in females (2.87 ± 0.35) with a significant sex × diet interaction (P_Int_ < 0.05) (Fig. 1E). Moreover, plasma free FA (FFA) and cholesterol concentrations were similarly increased in both the male and female HFD-fed groups (Fig. 1F, G). The results of the two-way ANOVA indicated a main effect of diet (P_Diet_ < 0.05) on plasma lipid concentrations, but no sex difference was observed. Because hepatic steatosis is a reliable marker of metabolic disorders in obesity models, we evaluated the liver steatosis score in all experimental groups (Fig. 1H, I). Significant lipid accumulation in the liver tissue was observed in HFD-fed male and female mice, as illustrated in Fig. 1H. Interestingly, HFD-fed female mice presented only a moderate increase in the hepatic steatosis score compared to males (Fig. 1I) with a significant sex × diet interaction (P_Int_ < 0.05), suggesting differential lipid accumulation in HFD-fed mice related to sex.

### Sex difference in renal function and structure in response to HFD

**Fig. 2:**
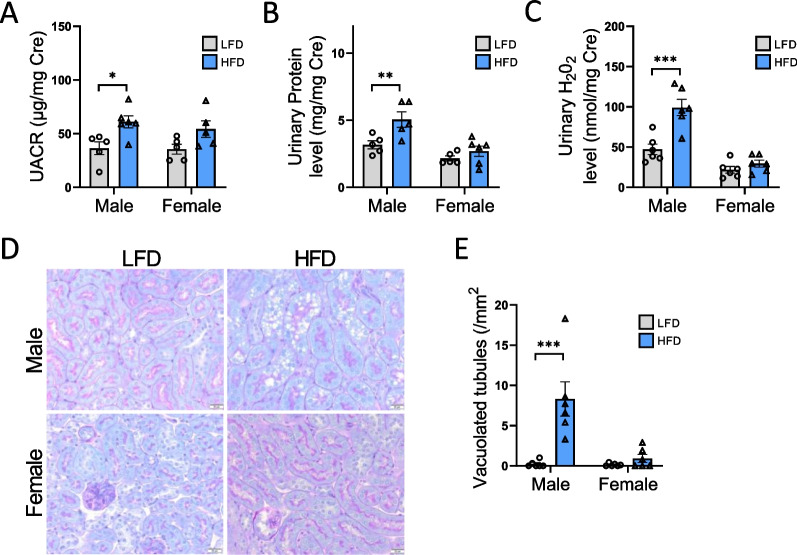
Histological changes and renal function in male and female mice fed a HFD. **A** Quantitative measurement of urinary albumin to creatinine ratio (UACR), **B** proteinuria and **C** urinary H_2_O_2_ level to creatinine ratio at week 16. **D** Representative photomicrographs (original magnification × 400) of PAS staining illustrating vacuolated proximal convoluted tubular cells from renal cortex section in males and females fed a LFD or a HFD. **E** Quantitative analysis of number of vacuolated tubules per mm^2^. Statistical analyses were performed using two-way ANOVA followed by Tukey post-test. *p < 0.05; **p < 0.01; ***p < 0.001. Data are presented as means ± SEM

Obesity-induced CKD is characterized by ectopic lipid accumulation in the S1 segment of PTC, leading to renal dysfunction [13]. Therefore, we first evaluated renal function by measuring urinary albumin (albuminuria) and protein (proteinuria) levels, both normalized to creatinine. In addition, urinary H_2_O_2_ level was measured as a marker of oxidative stress. As presented in Fig. 2A–C, albuminuria, proteinuria, and urinary H_2_O_2_ levels significantly increased in HFD-fed male mice. In female mice, only a slight increase (*p* = 0.072) in albuminuria was observed (Fig. 2A). Two-way ANOVA revealed that albuminuria was mostly affected by diet rather than sex (Table 2), whereas proteinuria and urinary H_2_O_2_ levels were significantly affected by sex with a significant sex × diet interaction for urinary H_2_O_2_ (P_Int_ < 0.005). Morphological changes in the renal cortex were also examined. The effects of sex and diet on proximal tubule morphology were analyzed (Fig. 2D). As expected, HFD-fed male mice displayed a high amount of vacuolized proximal tubules, as indicated by intracellular lipid droplet accumulation in proximal tubular cells. Surprisingly, female mice did not present any intracellular lipid droplets in the PTC when fed a HFD. These observations were confirmed by the quantification of the number of vacuolized tubules in each group (Fig. 2E). Two-way ANOVA analysis identified the effects of sex and diet, as well as a significant sex × diet interaction (P_Int_ < 0.005), suggesting a sex-specific effect on this parameter in HFD-fed mice.

### Sex-specific lipid accumulation in response to a HFD in the renal cortex

**Fig. 3:**
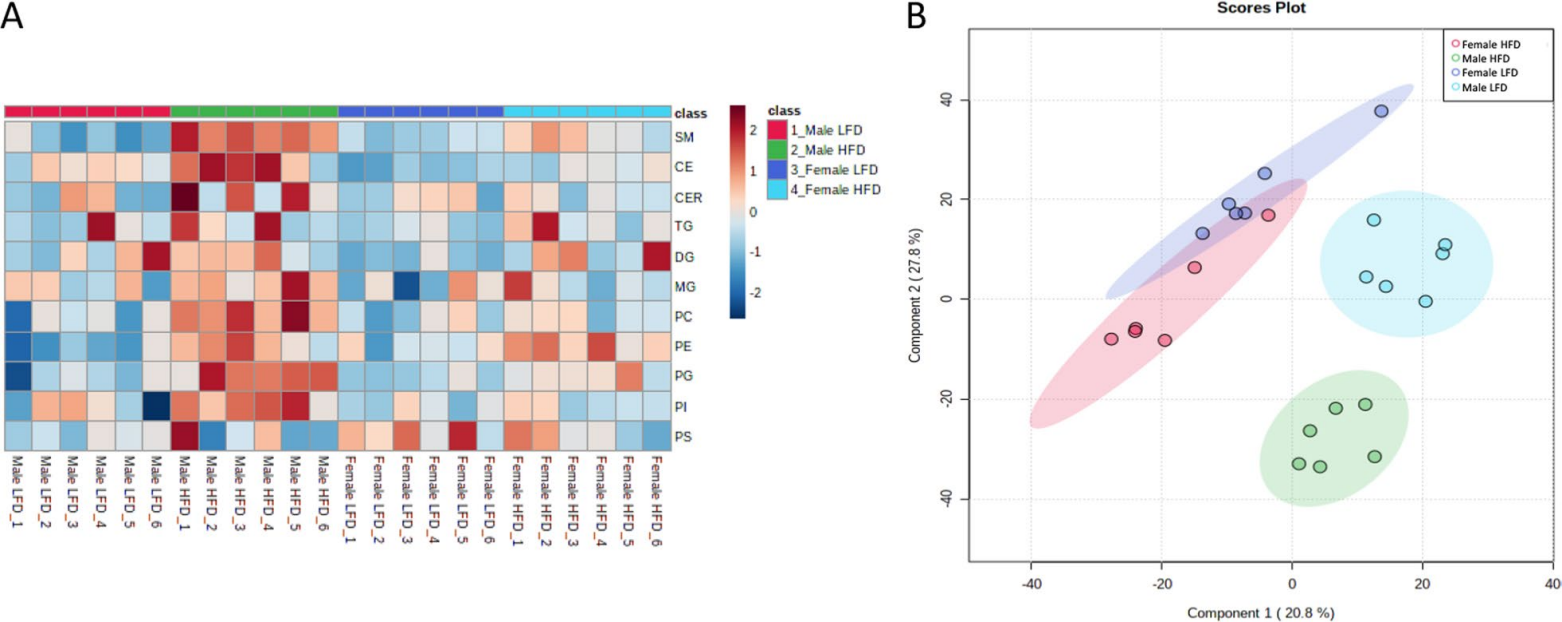
Sex differences in renal lipid in male and female mice fed a HFD. **A** Heatmap of lipidomic classes representing the relative differences in renal lipid accumulation between the groups. **B** 2D representation of the PLS-DA of renal lipid composition in LFD males (light blue), LFD females (dark blue), HFD males (green) and HFD females (red)

To further characterize the sex differences observed in renal lipid accumulation, targeted lipidomic analysis using MS was performed on renal tissues from males and females fed a LFD or HFD. As illustrated in Fig. 3A, the heat map presenting quantitative levels of lipid species (SM, CE, CER, TG, DG, MG, and phospholipids) for each animal demonstrates a strong accumulation of lipids in males fed a HFD for all lipid classes, whereas females seemed to be less affected by renal lipid accumulation following HFD feeding, which further confirms the absence of tubular vacuolization in HFD-fed females. To obtain an overview of the lipid species composition differences between sexes and dietary interventions, a PLS-DA was performed based on the relative amounts of lipid species for each animal. LFD and HFD-fed male mice formed separate clusters of lipid species, indicating different renal lipid compositions in response to diet, whereas HFD and LFD-fed female mice were not discriminated (Fig. 3B). Moreover, LFD-fed males and females presented separated clusters, suggesting a sex-specific composition of renal lipid species.

### Effect of HFD and sex differences on glycerolipids and CE in the renal cortex

**Fig. 4:**
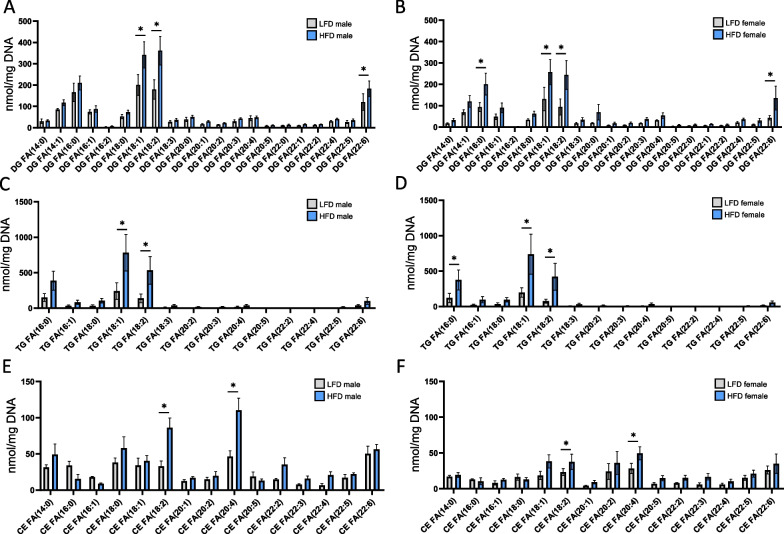
Sex differences in lipid content profiles (DG, TG, and CE) in renal cortex tissue from males and females in response to a HFD. Sum of the quantitative amount of FA species within a class after normalization (DNA content from tissue samples). **A, B** Changes in DG profile in males and females fed a HFD or a LFD. **C, D** Changes in TG profile in males and females fed a HFD or a LFD. **E, F** Changes in CE profile in males and females fed a HFD or a LFD. Statistical analyses were performed using multiple *t-*tests with *p* values corrected for multiple comparison using the Bonferroni method. Data are presented as means ± SEM. *n* = 4–6 in each group

To better investigate the effects of sex and diet on the kidney lipidome, lipidomic data for males and females were presented based on the number of double bonds and acyl chain length for each lipid class. Neutral lipid accumulation in kidneys is associated with diabetic nephropathy and obesity-induced CKD [20]. We evaluated DG, TG, and CE accumulation, as well as their FA-acid chain composition, in males and females fed a HFD. As illustrated in Fig. 4, both sexes displayed similar accumulation of glycerolipids (DG and TG). Analysis of acyl chain length and unsaturated bonds demonstrated the greatest accumulation of TG and DG with unsaturated 18:1 and 18:2 in HFD-fed mice. Moreover, DG 22:6 was also increased in HFD-fed male and female mice (Fig. 4A–D). Only females exhibited a significant increase in 16:0 for DG and TG lipid classes (Fig. 4B). Regarding sterol lipid accumulation, the data showed that renal CE content was lower in females than in males, even in lean animals (Fig. 4E, F). HFD induces increased levels of 18:2 and 20:4 CE, with a more marked accumulation in males. Collectively, these data demonstrate that males and females do not exhibit a drastic differential response in neutral lipid accumulation when fed a HFD.

### Sex-specific phospholipid accumulation in the renal cortex in response to a HFD and associated autophagy dysfunction

**Fig. 5:**
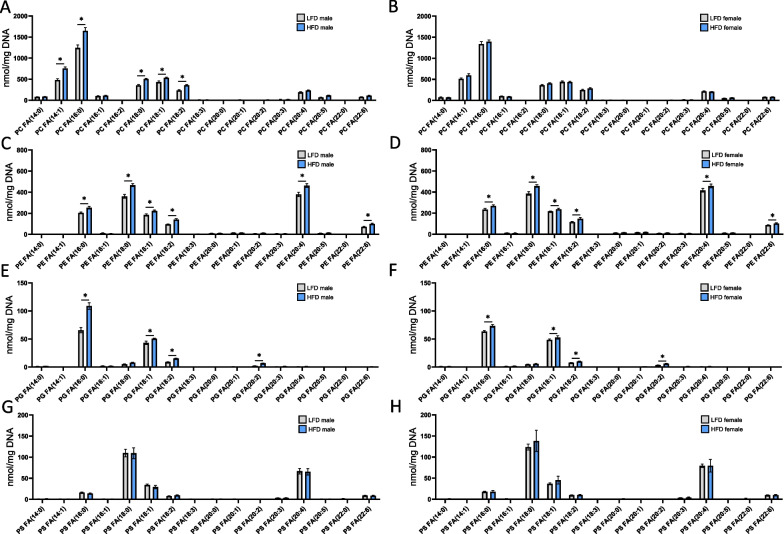
Sex differences in phospholipid content profile in renal cortex tissue from males and females in response to a HFD. Sum of the quantitative amount of FA species within a class after normalization (DNA content from tissue samples). **A, B** Changes in PC profile in males and females fed a HFD or a LFD. **C, D** Changes in PE profile in males and females fed a HFD or a LFD. **E, F** Changes PG profile in males and females fed a HFD or a LFD. **G, H** Changes PS profile in males and females fed a HFD or a LFD. Statistical analyses were performed using multiple t-tests with P values corrected for multiple comparison using the Bonferroni method. Data are presented as means ± SEM. *n* = 4–6 in each group

Using an in vitro model of renal lipid overload, Yamamoto et al. demonstrated impaired lysosomal accumulation and excessive accumulation of phospholipids originating from the plasma and organelle membranes [22]. Lipidomics analysis also revealed differences in phospholipid accumulation between the sexes and diets. As illustrated in Fig. 5A, B, females were remarkably protected from PC accumulation in the renal tissues. While LFD-fed males and females presented the same FA profile for PC, HFD males presented a significant increase in PC content, mostly for palmitic acid (16:0) but also 14:1, and fatty acids with 18C. In contrast, the FA profiles of other phospholipid classes, such as PE, PG, and PS, did not show any difference between males and females (Fig. 5C–H). Indeed, Fig. 5C, D shows the accumulation of PE in males and females in response to HFD. Both HFD-fed males and females accumulated PE 16:0, PE with 18C, 20:4 and 22:6 with similar fold changes between sexes.

**Fig. 6:**
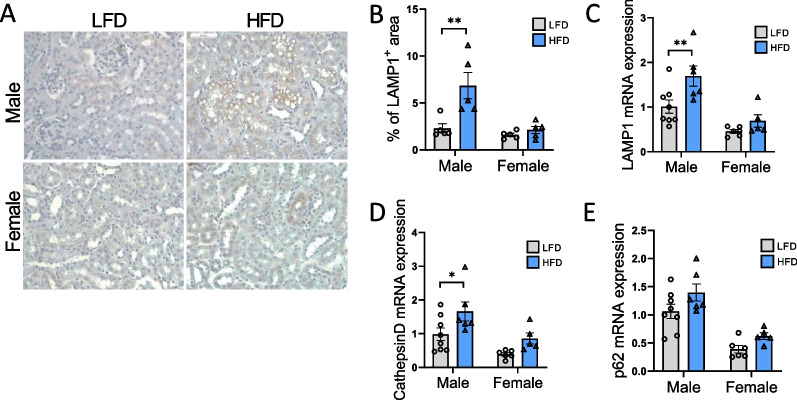
Sex differences in renal lysosomal dysfunction in males and females in response to a HFD. **A** Representative photomicrograph (original magnification × 40) showing LAMP1–positive staining on intracellular vacuoles in males and females fed a HFD or a LFD. **B** Related quantitative analysis of LAMP1-positive staining. **C–E** Real-time quantitative PCR analysis for LAMP1, Cathepsin D and p62 genes. mRNA expression was performed with kidney tissue from LFD and HFD female and male mice and normalized against 18S. Statistical analyses were performed using two-way ANOVA followed by Tukey post-test. *p < 0.05; **p < 0.01. Data are presented as means ± SEM

Studies suggest that HFD induces lysosomal storage and autophagy dysfunction in male mice, similar to the intra-lysosomal accumulation of phospholipids observed in PTC of animals and humans exposed to aminoglycosides or lysosomal storage diseases [13, 22, 41]. To assess this phenotype, we evaluated the abundance of the lysosomal marker LAMP-1 in renal tissues from male and female mice subjected to a LFD or HFD. A more pronounced LAMP-1 staining in renal cortex was observed in HFD-fed males, particularly in the PTC at the site of lipid droplet accumulation (Fig. 6A). The percentage of LAMP-1-positive area was significantly increased in HFD-fed males compared to LFD-fed males, but not in females with a significant sex × diet interaction (P_Int_ < 0.05), suggesting that lysosome accumulation in the renal cortex is sex-specific (Fig. 6B). Moreover, the expression of *Lamp1*, *Cathepsin D*, and *p62* was higher in males than in females, with a significant sex × diet interaction (P_Int_ < 0.05) for *Lamp1*, as shown in Fig. 6C–E. These data suggest the dysregulation of lysosomal markers (as described in [22]) in a sex-specific manner.

### Sex-specific CER accumulation in the renal cortex in response to HFD is associated with adiponectin and AMPK signaling

**Fig. 7:**
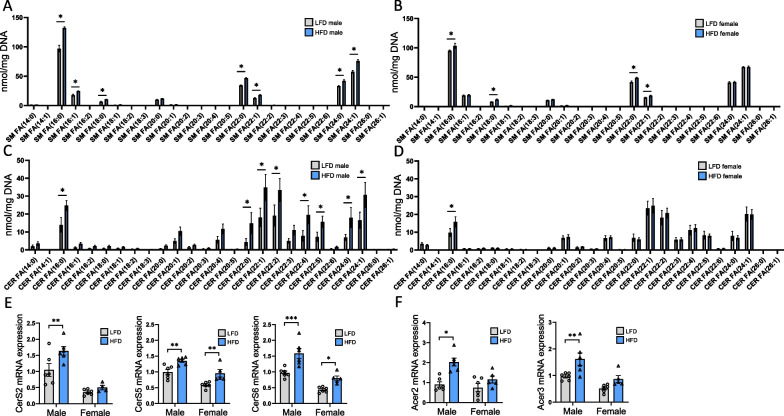
Sex differences in sphingolipid content profile and CER metabolism in renal cortex tissue from males and females in response to a HFD. Sum of the quantitative amount of FA species within a class after normalization (DNA content from tissue samples). **A, B** Changes in SM profile in males and females fed a HFD or a LFD. **C, D** Changes in CER profile in males and females fed a HFD or a LFD. Statistical analyses were performed using multiple t-tests with P values corrected for multiple comparison using the Bonferroni method. Data are presented as means ± SEM. *n* = 4–6 in each group. **E–F** Real-time quantitative PCR analysis for *CerS2*, *CerS5*, *CerS6*, *Acer2* and *Acer3* genes. mRNA expression was performed with kidney tissue from LFD and HFD female and male mice and normalized against 18S. Statistical analyses were performed using two-way ANOVA followed by Tukey post-test. *p < 0.05; **p < 0.01; ***p < 0.001. Data are presented as means ± SEM

Sphingolipids such as CER are mainly produced by the endoplasmic reticulum via de novo synthesis [42]. Among the sphingolipid classes, SM accumulation has been implicated in metabolic disturbances in diabetic nephropathy [43]. Male and female mice present differential renal accumulation of SM and CER species in response to HFD. While a strong accumulation of 16:0, 24:0, and 24:1 SM was observed in males, females only displayed moderate accumulation of 16:0 (Fig. 7A, B). CER accumulation in the kidneys is well known to contribute to renal disorders. Here, long chain (C16) and very-long-chain (22 to 24C) CER were particularly increased in HFD-fed male mice, while females exhibited a significant accumulation of 16:0 but no difference in very-long-chain CER content (Fig. 7C, D). The mRNA expression levels of CER synthases *CerS5* and *Cers6*, which predominantly produce C16-CER, were higher in LFD males than in LFD females. Their expression was significantly upregulated in both sexes fed a HFD (Fig. 7E, F). In contrast, the relative expression of *CerS2*, which produces CER incorporating 22- to 24-carbon FA, was only significantly increased in male HFD-fed mice. CER can be deacylated by a family of ceramidases that produce sphingosines to prevent lipotoxicity. Therefore, the expression of alkaline ceramidases *Acer2* and *Acer3* was quantified and was found to be upregulated in males but not in females in response to HFD.

**Fig. 8:**
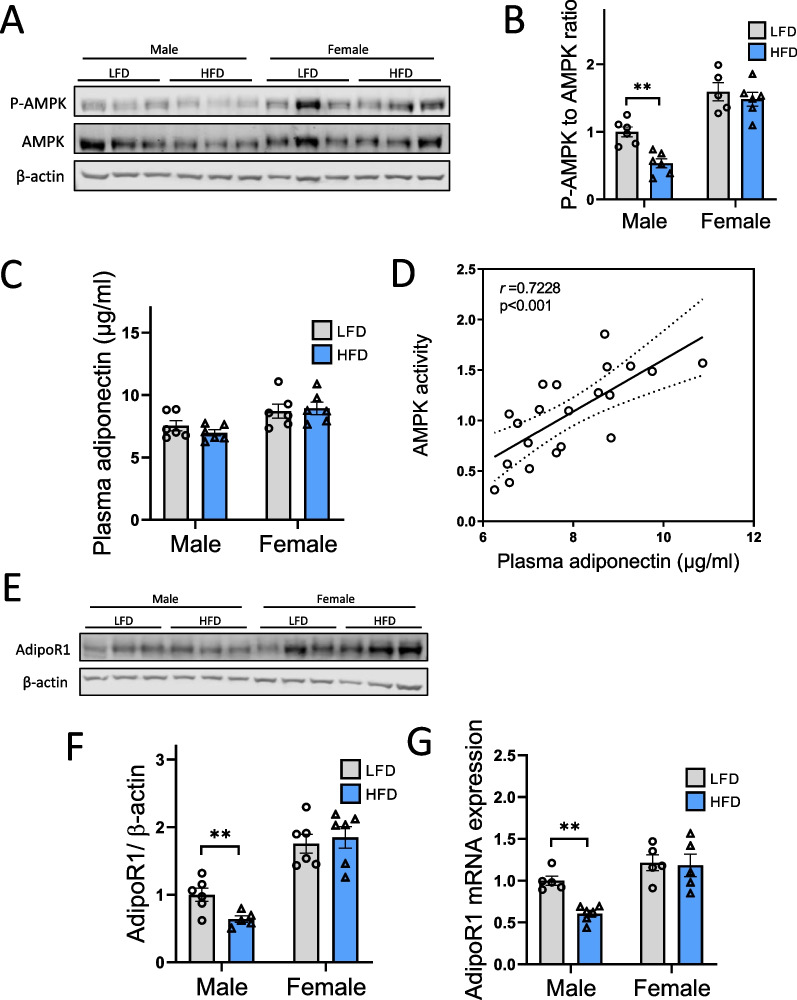
Sex differences in the renal AMPK/adiponectin pathway in males and females in response to a HFD. **A** Representative Western blotting of total and P-AMPK levels in renal cortex tissue. β-Actin was used as internal control. **B** Relative densitometry of the immunoblots representing P-AMPK to AMPK ratio in each group. β-actin was used to illustrate protein loading. **C** Plasma adiponectin level in males and females fed a HFD or a LFD at week 16. **D** Correlation between plasma adiponectin and AMPK activity (P-AMPK to AMPK ratio) for all animals. A significant positive linear correlation is present. **E** Representative Western blotting of AdipoR1 level in renal cortex tissue. β-actin was used as internal control. **F** Relative densitometry of the immunoblots representing AdipoR1 in each group. **G** Real-time quantitative PCR analysis for AdipoR1 gene. mRNA expression was performed with renal cortex tissue from LFD and HFD female and male mice and normalized against 18S. Statistical analyses were performed using two-way ANOVA followed by Tukey post-test. **p < 0.01. Data are presented as means ± SEM

An interesting link has recently been found between adiponectin levels and CER accumulation in HFD-fed mice, suggesting that adiponectin may protect against the accumulation of CER [44]. Therefore, based on our previous studies demonstrating that renal lipotoxicity in HFD-fed mice was associated with decreased AMPK activity, we further investigated the adiponectin–AMPK axis. Here, P-AMPK to AMPK ratios were first investigated in the kidneys of LFD and HFD male and female mice. The evaluation of AMPK activity in renal tissue by Western blotting was performed by calculating the ratio between phosphorylated (thr172) AMPKα and total AMPKα. As expected, a decreased P-AMPK:AMPK ratio was observed in HFD-fed male mice compared with LFD, reflecting a decrease in AMPK activity (Fig. 8A, B). Interestingly, female mice showed higher AMPK activity than males (P_Sex_ < 0.0001) without a decrease in P-AMPK:AMPK ratio when fed with HFD. Adiponectin stimulates the activation of AMPK via AdipoR1, and adiponectin-deficient mice show decreased AMPK phosphorylation [45]. In this regard, we evaluated the adiponectin/AMPK pathway in males and females. Plasma adiponectin levels were not significantly affected by HFD, but female mice presented higher levels of adiponectin than males (P_Sex_ < 0.005). This was significantly correlated with AMPK activity (phospho-AMPK to AMPK ratio) in renal tissues (Fig. 8D). Moreover, HFD selectively decreased AdipoR1 mRNA and protein expression in males (Fig. 8E–G). Taken together, these data suggest dysregulation of the adiponectin/AMPK pathway in HFD-fed males but not in females.

## Discussion

The U.S. National Institutes of Health (NIH) initiated recommendations to address over-reliance on males in preclinical research and improve sex balance [46]. Underrepresentation of female subjects in experimental and clinical research still results in a lack of understanding of the basic biological processes related to sex and poorer treatment outcomes for women [47]. Numerous studies have demonstrated that males and females differ in their physiology and metabolic homeostasis [48–50]. HFD-fed mice have been widely used as a suitable model to study obesity-related disorders [51]. While obesity-induced kidney disease is now well characterized in HFD-fed C57Bl/6J male mice, there are still no reliable data on the renal consequences of obesity in females. In this study, we explored the sex-specific effects of a HFD on kidney dysfunction and renal lipid accumulation. Indeed, we have previously demonstrated that dysregulated renal lipid metabolism and associated lipotoxicity play a major role in CKD development and progression in HFD-fed male mice [12–15]. Moreover, previous studies have demonstrated sex-specific lipid molecular signatures in the adipose tissue and liver of obese mice, contributing to the sex-dependent response to obesity [52]. Through targeted lipidomic analysis, we further characterized the renal lipid species composition, which may contribute to sexual dimorphism in renal disease development.

Despite similar BW gain and increased circulating leptin concentrations in both males and females, HFD feeding induced sex-related differences in metabolic parameters. Males but not females develop insulin resistance with hyperglycemia and hyperinsulinemia. This is consistent with other studies demonstrating that high caloric intake does not affect insulin sensitivity in female [53–55]. Importantly, female mice exposed to a prolonged period of HFD (36 weeks) have been associated with delayed metabolic dysfunction and insulin resistance compared to males [4]. Protection against obesity-associated insulin resistance was also decreased in ovariectomized females, suggesting that sex hormones play a crucial role in protecting female mice from metabolic disturbances induced by obesity [56]. Clinical studies have also demonstrated that diabetes is more prevalent in obese men than in obese women [57]. However, menopause has been associated with detrimental effects on glucose homeostasis, while estrogen-based hormonal therapy protects against the development of diabetes [58]. There is clear evidence in clinical and experimental studies that the development of insulin resistance in obese individuals is influenced by sex and affects more men than women [57]. However, the relationship between sex and obesity-related disorders, such as dyslipidemia, ectopic lipid accumulation, and lipotoxicity, remains ambiguous. Dyslipidemia is a common feature of obesity, and is associated with diabetes and fatty liver disease [59]. In the present study, both male and female HFD-fed mice presented increased plasma lipid levels, with a more pronounced increase in plasma FFA in females, whereas males showed higher levels of plasma cholesterol. Studies have shown that obesity increases plasma FFA levels in both sexes due to increased lipolysis in adipose tissue [60]. However, female animals as well as women are protected from FFA-induced insulin resistance [61, 62]. Experimental data from animal models suggest that estrogens protect insulin-sensitive tissues from insulin resistance by activating the ERα pathway in females [63]. Many studies have reported sex differences in diet-induced non-alcoholic fatty liver disease development, with varying outcomes depending on the strain or diet composition [64–67]. Here, increased plasma lipid levels were associated with hepatic steatosis in both the sexes. However, hepatic steatosis was more pronounced in males fed a HFD than in females, which is relevant compared to experimental and epidemiological studies [67, 68].

Epidemiological studies have found that sex and gender differences exist in the susceptibility, prevalence, and progression of CKD [10]. Although premenopausal women appear to be protected from non-diabetic renal diseases, the incidence of CKD is higher in women than in men, whereas progression to end-stage renal disease is more common in men [69]. Many experimental models of kidney diseases have demonstrated that sex hormones in females exert renoprotective effects [70–72]. Regarding obesity, the relationship between body mass index and CKD also seems to differ according to gender [73]. The lipid metabolism of males and females significantly differs, which may impact adaptation to lipid overload in targeted organs during obesity, including the kidneys [50].

In the present study, we showed that female mice fed a HFD were protected from renal function impairments. Indeed, hallmarks of renal dysfunction, such as increased albuminuria, proteinuria, and even increased levels of urine hydrogen peroxide, were significantly elevated in obese male mice, but not in females. We and others have previously demonstrated that the proximal tubule is the primary site of lipid deposition during obesity [13, 14, 22]. Concentric membrane layer structures (multilamellar bodies) as well as cytoplasmic neutral lipid droplets have been found in murine models of obesity-induced CKD, as well as in patients [13, 20, 22, 74]. In particular, our previous study demonstrated that the proximal tubular epithelium of male mice fed a HFD accumulated large lipid vacuoles [12]. Interestingly, in the current study, no vacuolization was detected in the renal cortex of the female mice. Therefore, to better link renal physiopathology to lipid species composition between males and females, a targeted lipidomic approach was used. Although both male and female mice fed a HFD exhibited renal lipid accumulation, male mice were found to be more affected than females for all lipid species, with a drastic change in renal lipid composition.

Importantly, lipidomic analysis showed that females do accumulate specific lipid species when fed with HFD, despite the absence of vascularized tubules in renal cortex sections. This is particularly true for neutral lipids, such as DG, TG, and cholesterol. Indeed, a very similar accumulation of DG and TG, with mostly side chains of oleic (18:1) and linoleic (18:2) acids, was found in both males and females fed a HFD. In addition, kidneys of obese mice mostly accumulate CE with linoleic and arachidonic acid, while males present higher levels of CE compared to females. This suggests that HFD feeding leads to renal lipid accumulation in both sexes but not necessarily lipotoxicity. Indeed, lipotoxicity is a consequence of an imbalance between FA uptake, utilization (notably through beta-oxidation), and storage. Storage relies on esterification of a glycerol backbone to form TG, which is then stored within lipid droplets. Triglycerides themselves are not toxic but serve as an active reservoir of FA as a direct consequence of lipid overload. Renal cells are known to have an important capacity to store TG as lipid droplets, notably during fasting, in both males and females [75]. Increased serum NEFA levels are directly associated with increased TG accumulation in the kidneys. Thus, increased lipid storage in the kidneys of male and female mice could be the consequence of elevated plasma levels of NEFA found in both sexes when fed with HFD. Interestingly, increased levels of DG were not associated with kidney impairment in females, suggesting that DG intermediate accumulation in the kidney could not be particularly linked to direct lipotoxicity.

Besides, caloric excess leads to upregulation of the de novo CER synthesis pathway [76]. CER play an important role as lipotoxic mediators of metabolic dysfunction in fatty liver disease, cardiovascular diseases, and diabetes [77]. Differences in circulating CER profiles have been significantly associated with the development of macroalbuminuria and CKD in humans [78]. However, the relevance of CER metabolism in kidneys remains unclear. Here, we demonstrated sex differences in the abundance and composition of CER lipid species. While HFD-fed male mice displayed a strong accumulation of long chain (C16) as well as very-long-chain (C22–C24)-CER, females only showed a moderate but still significant increase in C16-CER levels. Numerous studies have suggested that specific CER species are more relevant in pathological events than changes in total CER concentrations, although the exact role of each CER species is still debated [76]. Law et al*.* demonstrated that very long-chain CER, but not long-chain (C16 and C18) CER, causes mitochondrial dysfunction and cell death in cardiomyocytes [79]. In our study, only male HFD-fed mice presented an increased level of very-long-chain CER and exhibited evidence of renal dysfunction (as attested by marked albuminuria, proteinuria, and urine hydrogen peroxide). Moreover, the expression of *CerS2*, which produces very long-chain (C20–26) CER species, was only enhanced in male mice fed a HFD. In contrast, the expression of *CerS5* and *Cers6*, which promote the incorporation of long-chain (e.g., C16) acyl groups, was increased in both sexes and is related to increased levels of C16-CER. Collectively, these data suggest that HFD-fed females are protected from very-long-chain CER accumulation in the kidneys and their associated lipotoxic effects. Recently, a link between adiponectin axis and CER metabolism was highlighted, suggesting that adiponectin, through its interaction with its receptors, induces the activation of ceramidases that lead to beneficial effects on mitochondria in adipocytes and hepatocytes [26]. Adiponectin is a 30-kDa circulating plasma protein that is primarily secreted by adipocytes. Adiponectin can bind to three receptors: AdipoR1, -2 and T-cadherin. Adipo-R1 and -R2 have anti-atherogenic, anti-inflammatory, and hypoglycemic properties [80]. Here, even though genic ceramidase expression was not increased in females, plasma adiponectin levels as well as mRNA and protein levels of the adiponectin receptor were higher in HFD-fed females than in males. Adiponectin is a key regulator of glucose and lipid metabolism in the kidneys [81]. This adipokine is involved in the reduction of inflammation, fibrosis, and oxidative stress through activation of AMPK and peroxisome proliferator-activated receptor alpha (PPARα) [82]. Besides, it has been demonstrated that AMPK inhibits de novo CER synthesis in skeletal muscle and astrocytes [83, 84]. AMPKα1 deficiency was also associated with lipid droplet accumulation in the kidney tubules and increased CER accumulation [85]. Moreover, we previously demonstrated the crucial role of AMPK in renal cell dysfunction [13, 14]. Female mice were protected from decreased AMPK activity, which correlated with higher circulating levels of adiponectin in females. Studies using AdipoR1 knockout mice showed decreased AMPK activity, while AMPK activity correlated with adiponectin levels in an obesity model [45, 86]. Moreover, treatment of db/db mice with an activator of adiponectin, AdipoRon, resulted in upregulation of phosphorylated AMPK in the kidney along with reduced inflammation and lipotoxicity [26]. Thus, a dysregulated adiponectin/AMPK pathway in HFD-fed males could lead to CER-induced lipotoxicity and renal injury. AMPK plays a key role in the regulation of autophagy in mammalian cells [87]. Alterations in autophagy, lysosomal dysfunction, and lysosomal lipid storage have been described as the hallmarks of renal lipotoxicity [3]. Yamamoto et al. demonstrated the accumulation of dysfunctional lysosomes containing phospholipids of plasma and organelle membrane origin using an in vitro model of renal lipid overload [22]. In the present study, lipidomic analysis revealed significant accumulation of PC species in the kidneys of HFD-fed male mice. This change was associated with an increase in the number of lipid vacuoles in proximal tubules that were positive for lysosomal markers. Therefore, these results are in line with those of Yamamoto et al. and confirm that the intra-lysosomal lipid storage found in males is likely composed of phospholipids [22]. Moreover, consistent with our previous studies, our results showed that maintaining AMPK activity, as observed in female mice, is associated with preserved tubular homeostasis.

### Perspectives and significance

Our study revealed that renal lipid species signatures in mice in response to HFD are markedly different between sexes. Our results showed that females are protected from the accumulation of very long CER, which might contribute to renal lipotoxicity in males. To further advance our understanding of the specific role of CER metabolism in response to HFD, future studies targeting ceramide synthase and ceramidase in the mouse kidney would be highly valuable. Examining ceramidase activity in the kidney could also provide additional insights into the mechanisms underlying the protection of female mice from very-long-chain ceramide accumulation. In addition, only males presented a strong accumulation of phospholipids related to autophagy and lysosomal impairment. Furthermore, we explored the putative link between the adiponectin–AMPK axis and the prevention of lipotoxicity and CKD development in females. Further studies are warranted to delineate the precise role of adiponectin in female protection against renal lipotoxicity. Indeed, it cannot be excluded that the sustained AMPK activity in obese females may be attributed to factors other than adiponectin and that the low levels of toxic lipids such as ceramides may play a beneficial role. Thus, the use of adiponectin knockout female mice on HFD would be an interesting mechanistic strategy to confirm the adiponectin/AMPK axis in renal tissue. Moreover, studies using human samples such as renal biopsy of diabetic/obese patients as well as related clinical data are needed to enhance the clinical relevance of our findings and provide valuable insights in the sexual dimorphism of obesity-induced renal lipid accumulations.

This novel characterization might contribute to the understanding of functional differences in obesity-induced CKD between males and females.

## Data Availability

Please contact author for data requests.
